# Metrology part 1: definition of quality criteria

**DOI:** 10.1007/s10877-020-00494-y

**Published:** 2020-03-17

**Authors:** Pierre Squara, Thomas W. L. Scheeren, Hollmann D. Aya, Jan Bakker, Maurizio Cecconi, Sharon Einav, Manu L. N. G. Malbrain, Xavier Monnet, Daniel A. Reuter, Iwan C. C. van der Horst, Bernd Saugel

**Affiliations:** 1 Department of Cardiology and ICU, Clinique Ambroise Paré, Neuilly-sur-Seine, France; 2 Department of Anesthesiology, University of Groningen, University Medical Centre Groningen, Groningen, The Netherlands; 3 Intensive Care, St Georges’ University Hospitals NHS Foundation Trust, London, UK; 4 Departmento de Medicina Intensiva, Facultad de Medicina, Pontificia Universidad Católica de Chile, Santiago, Chile; 5 Department of Intensive Care Adults, Erasmus MC University Medical Center, Rotterdam, The Netherlands; 6 Department of Pulmonary and Critical Care, New York University, New York, USA; 7 Division of Pulmonary, Allergy, and Critical Care Medicine, Columbia University Medical Center, New York, USA; 8 Department of Anesthesia and Critical Care, Humanitas University, Milan, Italy; 9 General Intensive Care Unit of the Shaare Zedek Medical Centre, Hebrew University Faculty of Medicine, Jerusalem, Israel; 10 Department of Intensive Care, University Hospital Brussels (UZB), Jette, Belgium; 11 Faculty of Medicine and Pharmacy, Vrije Universiteit Brussel (VUB), Brussels, Belgium; 12 Medical Intensive Care Unit, Paris-Sud University Hospitals, Assistance Publique-Hôpitaux de Paris, Inserm UMR S_999, Le Kremlin-Bicêtre, France; 13 Department of Anesthesiology and Intensive Care Medicine, University Medical Center Rostock, Rostock, Germany; 14 Department of Intensive Care, Maastricht University Medical Center+, Maastricht University, Maastricht, The Netherlands; 15 Department of Anesthesiology, Center of Anesthesiology and Intensive Care Medicine, University Medical Center Hamburg-Eppendorf, Hamburg, Germany; 16 Outcomes Research Consortium, Cleveland, OH USA

**Keywords:** Statistics, Critical care, Perioperative medicine, Hemodynamic monitoring, Cardiovascular dynamics

## Abstract

Any measurement is always afflicted with some degree of uncertainty. A correct understanding of the different types of uncertainty, their naming, and their definition is of crucial importance for an appropriate use of measuring instruments. However, in perioperative and intensive care medicine, the metrological requirements for measuring instruments are poorly defined and often used spuriously. The correct use of metrological terms is also of crucial importance in validation studies. The European Union published a new directive on medical devices, mentioning that in the case of devices with a measuring function, the notified body is involved in all aspects relating to the conformity of the device with the metrological requirements. It is therefore the task of the scientific societies to establish the standards in their area of expertise. Adopting the same understandings and definitions among clinicians and scientists is obviously the first step. In this metrologic review (part 1), we list and explain the most important terms defined by the International Bureau of Weights and Measures regarding quantities and units, properties of measurements, devices for measurement, properties of measuring devices, and measurement standards, with specific examples from perioperative and intensive care medicine.

## Introduction

“Metrology is the science of measurement, embracing both experimental and theoretical determinations at any level of uncertainty, in any field of science and technology” [1]. Metrology is therefore of key importance not only for engineers and scientists but also for any clinician using tools to measure, assess, or estimate physiological variables. Understanding metrological concepts and recognizing their limitations and constraints is a prerequisite for the interpretation of data obtained within clinical practice and research, especially research on the validation of new medical devices.

Unfortunately, there is still a lot of confusion regarding the exact definitions of metrological terms in many papers reporting the results of method comparison studies. For example, most papers claim to report the “accuracy” of a measuring instrument despite the fact that accuracy qualifies a single measurement and cannot qualify a measuring instrument.

In addition, the new directive on medical devices of the European Union [2] mentioned that the notified body needs to be involved in all aspects related to the conformity of the device with the metrological requirements. Therefore, physicians need to adopt the appropriate terms and definitions as a first step before determining a set of minimum metrologic requirements for any measuring instrument used in perioperative and intensive care medicine.

Consequently, physicians must rigorously share the same terms and definitions with other scientists. This is of particular importance in perioperative and intensive care medicine because clinical decision-making considers, or even completely relies on a variety of variables, measured using medical devices including advanced hemodynamic and respiratory monitoring.

The consensual metrological list of terms of the "International Vocabulary of Metrology (VIM)" is divided into five main headings: (1) quantities and units, (2) measurement, (3) devices for measurement, (4) properties of measuring devices, and (5) measurement standards (Etalons) [1]. The complete list can be found in a guidance document of the Joint Committee for Guides in Metrology [1].

In the present document (part 1), we describe and define terms used to qualify and quantify medical measurements to provide a framework for a common and standardized way of describing, reporting, and discussing measurements in perioperative and intensive care medicine.

## Quantities and units

A quantity is a property of a phenomenon, body, or substance, to which a magnitude is attributed, that can be expressed as a number and a reference (a measurement unit, a measurement procedure, a reference material, or a combination of such). A quantity is, therefore, characterized by a dimension, a unit, and a value. There are seven base quantities from which all quantities of the international system (SI) are derived. They are listed in Table 1 together with five derived quantities frequently used in medicine. The complete list can be found in documents released by the intergovernmental organization International Bureau of Weight and Measures (BIPM) [1]. In assessing quantities, the VIM distinguishes facts (measurements) and methods (instruments).

## Measurement

A **measurement** is a process of experimentally obtaining one or more values that can reasonably be attributed to a quantity [1]. Since the true value of a quantity is necessarily unknown, a measurement result is generally expressed as a value and a measurement uncertainty. The **measurand** is the quantity to be measured [1]. A **measurement method** is based on a **measurement principle**, i.e., a physical, chemical, or biological phenomenon serving as the basis of a measurement [1]. A **reference measurement procedure** is a measurement procedure that provides measurement results fitting for their intended use [1]. Although it has no international definition, a **criterion standard** (often referred to as **gold standard)** is supposed to be the best practically available reference method. A **measurement error** is the difference between a single measurement and a reference quantity value.

**Fig. 1:**
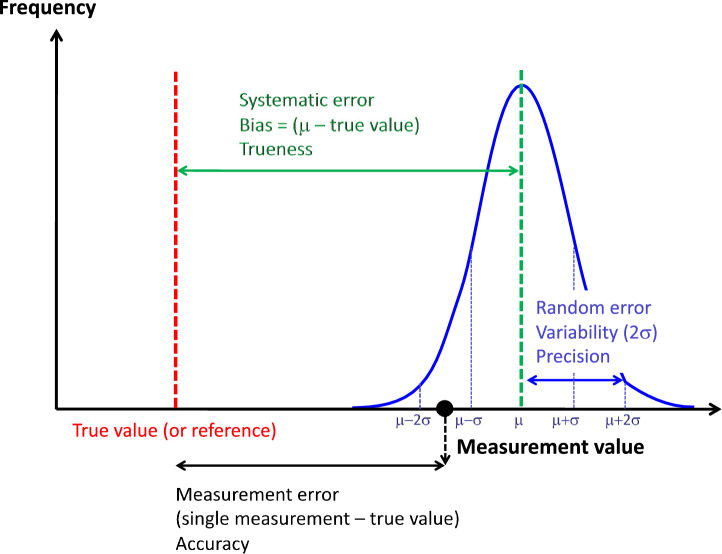
Schematic representation of the different types of measurement errors with an indication of the formula by which it is derived and the corresponding quality criteria. The black point represents a single measurement value, the blue curve is the frequency distribution of the values in case of replicate measurements of the same object under the same conditions, µ = mean, σ = standard deviation. Reproduced from [7] with permission

The uncertainty of a measurement is characterized by different components listed below and schematized in Fig. 1.

### Measurement precision

**Fig. 2:**
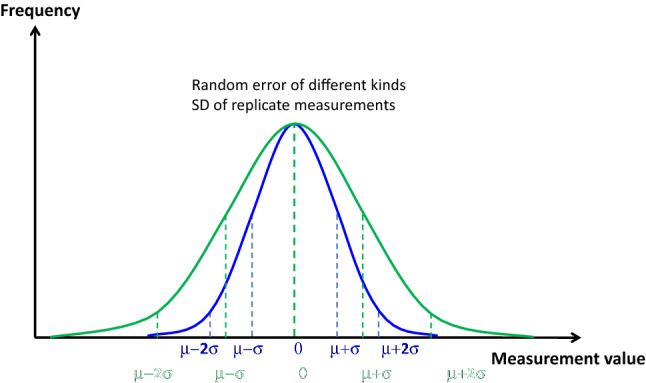
Schematic representation of the different types of precision. The blue distribution shows the smallest random variability (repeatability) for replicate measurements of the same quantity. The green distribution shows the largest variability (reproducibility). The variability corresponding to intermediate precision lies between the blue and the green curves. The average of random errors is zero

The **precision** is the closeness of agreement between measured values obtained by replicate measurements on the same or similar quantities under specified and stable conditions [1, 3]. In other words, precision describes the variability of replicate measurements of a given quantity value, without reference to a true or reference value (Fig. 1). Precision is a quality and should not be expressed as a numerical value but is generally assessed by **the random measurement error**. The random measurement error can be expressed as a number by the standard deviation (σ) or variance (σ^2^) of the repeated measurements and assuming a mean random error of zero (Figs. 1, 2). The coefficient of variation (2σ/mean) can also be expressed as variability in %. The specified conditions of precision assessment may add variabilities of different kinds [3]. **Repeatability** is the precision under conditions that include the same measurement procedure, same operators, same measuring system, same operation conditions, same location, and replicate measurements on the same or similar objects over a short period of time [1]. **Reproducibility** is the precision under a set of conditions that include different locations, operators, measuring systems, and replicate measurements on the same or similar objects [1]. Between repeatability and reproducibility, **intermediate precision** is the precision under a set of intermediate conditions of a measurement (Fig. 2) [1].

#### *Example *

If the systolic blood pressure of a patient, measured by a pressure transducer connected to a radial arterial catheter, is constant for 20 min (showing a stable reference quantity value irrespective of its value), and if 20 consecutive oscillometric upper-arm cuff measurements fluctuate during the same time between 110 and 130 mmHg (σ = 12.5 mmHg), the oscillometric upper-arm cuff measurement can be described as being “precise” or “imprecise”, according to the intended use if this level of variability is considered as excessive or acceptable.

### Measurement trueness

The **trueness** is the closeness of agreement between the average of an infinite number of replicate measured quantity values and the true or reference quantity value of the measurand [1]. Trueness is a quality and cannot be expressed as a numerical value but is generally assessed by **the systematic measurement error** [1]. Since the mean random error of an infinite number of replicates is zero, the difference between the averaged measured value and the reference value (also called **measurement bias**) is, therefore, an estimate of the systematic measurement error (Fig. 1). Consequently, a measurement with a small systematic measurement error is considered to be true [1]. A **correction** can be applied to compensate for a known systematic error.

#### *Example*

If the radial arterial catheter-derived systolic blood pressure of a patient is constantly measured at 120 mmHg over 20 min (stable reference quantity value) and if averaging 20 consecutive oscillometric upper-arm cuff measurements during the same time give a mean value of 120 mmHg, the systematic measurement error (measurement bias) is therefore 0. The oscillometric measurement mean value can be described as being true, irrespective of its variability.

### Measurement accuracy

The **measurement accuracy** is the closeness of agreement between a single measured value and a true or reference value of the measurand [1]. Accuracy is a quality and cannot be expressed as a numerical value but is generally assessed by a **measurement error** (Fig. 1) [1]. A measurement with a small measurement error is considered accurate [1]. A measurement error can, therefore, be the result of a random measurement error (σ; qualifying the imprecision), a systematic measurement error (bias, qualifying the untrueness), or both [1].

#### *Example*

If a single radial arterial catheter-derived systolic blood pressure measurement is 120 mmHg (reference quantity value) and if a single simultaneously obtained oscillometric upper-arm cuff measurement is 150 mmHg, the oscillometric upper-arm cuff measurement error is 30 mmHg and combines random and systematic measurement errors. This measurement can be described as being "inaccurate” according to the intended use and combines untrueness and imprecision.

### Measurement uncertainty

In the error approach (traditional approach, see above) the measurement error adds systematic and random errors, but no rule can be derived on how they combine for any given measurement. The **uncertainty** approach aims at characterizing the dispersion (pattern of distribution) of the values being attributed to a measurand, based on the information used [1]. This concept is broader than precision and may add systematic effects including uncertainty due to the reference method, time drift, definitional uncertainty, and other uncertainties. The objective of measurement in the uncertainty approach is not to determine a true value as closely as possible, but to reduce the range of values that can reasonably be attributed to the measurand [1].

**Note**

The translation from one language to another may be another source of confusion. For example, the VIM [1] is written in French and English. The French translation of “precision” is “fidélité” whereas “fidelity” in English is not mentioned in the document and usually refers to the degree of exactness with which something is copied or reproduced.

One solution would be to ban these quality concepts (accuracy, trueness, and precision) for which no specified numerical values are given and to be descriptive, speaking of “measurement error”, “systematic measurement error”, and “random measurement error”.

This—for two main reasons—is especially the case when using Bland-Altman analysis: First, the Bland-Altman plot has been proposed to compare two measuring instruments “when neither provides an unequivocally correct measurement” [4]. The second reason is that one important condition for estimating systematic and random errors is to average replicate measurements of the same quantity. Therefore, when several intra- or inter-patient measurements are done under different conditions, these estimations are strictly speaking impossible [3], and the criterium that is studied is the systematic discordance (or difference in agreement) between the two measuring instruments and its variability under different conditions.

An appropriate use of the Bland-Altman analysis to estimate the measurement trueness and precision would require: (1) a reference method and (2) replicate measurements of the same quantity, for example replicate measurements of the same measurand in the same patient in steady-state conditions (see part 2).

## Methods (instruments for measurements)

A **measuring instrument** is a device used for making quantity measurements, alone or in conjunction with one or more supplementary devices (measuring system) [1]. A measuring instrument is frequently a **transducer**, i.e., a device that provides an output quantity (most often an electric signal) having a specific relation with an input quantity (most often a physiological signal). The physiological signal is collected by a **sensor** defined as an element of a measuring system that is directly affected by a phenomenon, body, or substance carrying a quantity to be measured, or less frequently by a **detector** defined as a device or substance that indicates the presence of a phenomenon, body, or substance when a threshold value of an associated quantity is exceeded [1].

## Properties of measuring instruments (or devices)

An **indication** is a value provided by a measuring instrument [1]. An indication may result from many elementary measurements followed by a mathematic and/or algorithmic treatment. The **measuring interval** (or measuring range) is the set of values of the same kind that can be measured by a given instrument with specified instrumental uncertainty, under defined conditions [1]. A measuring instrument/system is characterized by different properties. The three main qualities of measurements seen before (precision, trueness, and accuracy) are obviously linked to instrumental properties, however, although measurements are facts that cannot be changed, instrumental properties are methods that can be improved by specific interventions (Table 2). Yet, the daily use may create confusion even in scientific documents. This is another reason for better being descriptive.

**Table 1:** International system of units

Quantity	Dimension	Unit	Symbol
Length	L	Meter	m
Mass	M	Kilogram	kg
Time	T	Second	s
Current	I	Ampere	A
Temperature	Θ	Kelvin	K
Amount of substance	N	Mole	mol
Luminous intensity	J	Candela	cd
Force	ML T^−^^2^	Newton	N
Pressure	ML^− 1^T^− 2^	Pascal	Pa
Work or energy	ML^2^T^− 2^	Joule	J
Power	ML^2^T^− 3^	Watt	W
Frequency	T^− 1^	Hertz	Hz

All other quantities can be derived from these base quantities such as flow = volume (length^3^) / time (L^3^T^-1^) or hydraulic resistance = pressure/flow (ML^−4^T^−1^)

**Table 2:** Summary of measurement qualities and instrumental properties

**Measurements (facts)**			
Quality	Quantity	Numerical value	Correction
Measurements precision	Random error	MV: σ, 2σ, 2σ/mean	–
Measurements trueness	Systematic error	Bias = AMV - R	–
Measurement accuracy	Measurement error	MV-R	–

**Instruments (methods)**			
Property	Quantity	Numerical value	Correction
Instrumental precision	Random error	IV: σ, 2σ, 2σ/mean	Signal/noise
–	Systematic error	Bias = AIV-R	Zero, offset
Sensitivity		ΔIV/ΔR	Signal, gain
Linearity		ΔIV/ΔR = constant	Signal, gain
Resolution/step time response		Linked to SEM of IV	Signal, gain

*MV *measurement value, *IV * indication value, *R * reference value, *A *average, *Δ * change, *SEM * standard error of the mean

### Instrumental precision (sometimes called precision of method)

In analogy to the measurement precision, the instrumental precision is the closeness of agreement between indications obtained by replicate measurements on the same or similar quantities under specified and stable conditions [1, 3]. Although this is incorrect, the quality “instrumental precision” is often confounded with its linked “quantity”, the variability of indications.

#### *Example*

If the real blood flow of a patient is stable and equal to 5 L/min (as produced by a calibrated pump, or measured using a reference method such as an internal flow probe) and if, at the same time, 20 consecutive indications of a measuring device vary from 4.7 to 5.3 L/min, the random error of the indications can be estimated by σ = 0.25 L/min, 2σ = 0.5 L/min, or 2σ/mean value = 10%. Whether it can be said that the instrument precision is acceptable or not depends on the intended use. Strictly speaking, it should not be concluded that the instrumental precision is 10%.

### Instrumental bias

In analogy to the measurement bias, the **instrumental bias** is the average of replicate indications minus a reference quantity value [1]. It estimates the systematic error provided by the measuring device. There is no quality linked to the instrumental bias, such as “instrumental trueness” in the VIM. Since accuracy is qualifying one single measurement, this quality cannot be used to describe an instrument. However, the term **“accuracy class”** is used to qualify measuring instruments that meet stated metrologic requirements.

#### *Example*

If the real blood flow of a patient is stable at 5.0 L/min (as measured in the example in 5.1), and if, at the same time, the average of 20 consecutive indications of a measuring device is 6.0 L/min, the tested measuring device has an instrumental bias of 1.0 L/min.

### Sensitivity

**Fig. 3:**
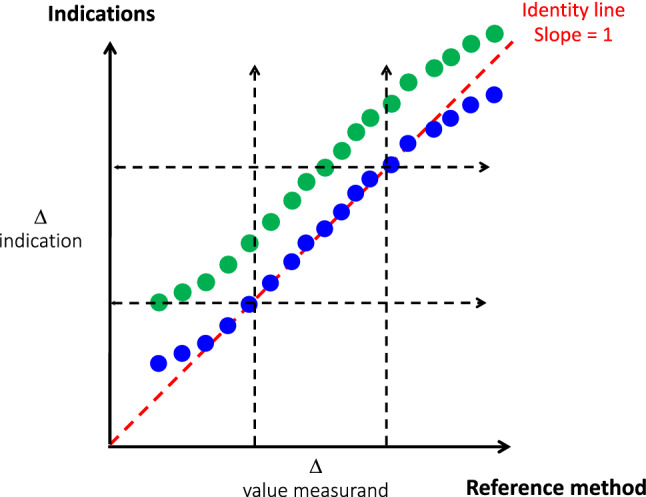
Schematic representation of the sensitivity. The blue points represent the indications of a device when the measurand is increasing. Within the range figured by the dotted arrows (measuring interval), the sensitivity is good and constant (linearity close to the identity). Under and over this interval, the sensitivity/linearity is altered with over- and underestimation of the true changes, respectively. The green points represent the indication of another device with the same sensitivity but with a positive instrumental bias

The **sensitivity** is the quotient of the change in an indication and the corresponding change in a measurand [1]. The change considered must be large compared with the resolution (defined below, Fig. 3) [1]. The metrological sensitivity should not be confounded with the statistical sensitivity. Being a quotient between two changes, sensitivity is mathematically a regression slope, ideally = 1. **Linearity**, which is not a metrological but a mathematical property, illustrates the property of maintaining the sensitivity constant over the measuring interval. In other words, the linearity is also the capability of maintaining the instrumental bias constant. Preferably, the regression line should be close to the identity line (y = x, bias = 0 on the measuring interval). When the slope is not on the identity line (y = ax; a ≠ 1), it shows a constant but poor sensibility. When the slope formula is (y = ax + b; b ≠ 0), the sensibility can be good in a part of the measuring interval but not on the whole as exemplified in Fig. 3.

#### *Example*

If the real blood flow (as measured in the example in 5.1) is changing from 4.0 to 6.0 L/min, and if, at the same time, the indications of a measuring device change from 4.5 to 5.5 L/min, although the mean values are comparable, the tested measuring device is not sensitive.

### Selectivity

**Fig. 4:**
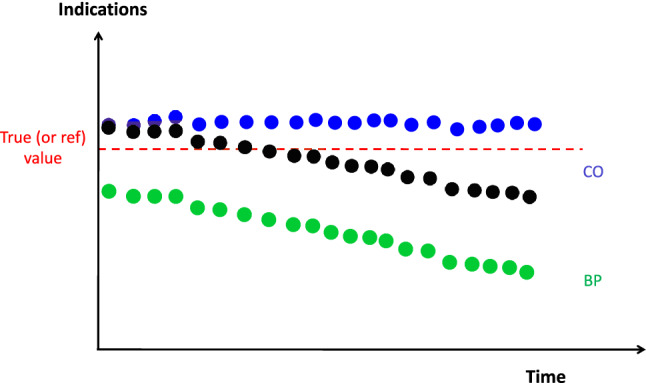
Schematic representation of the selectivity. In this example, the indications from two different devices for cardiac output assessment (CO 1; blue points and CO 2; black points) are collected when blood pressure (BP; green points) is decreasing while the true CO is maintained constant (red line). The CO 1 device, although systematically overestimating the true CO, is selective since indications are independent of the BP. The CO 2 device, although assessing CO more truly at the onset of the test, is not selective since its indications covary with BP

The **selectivity** is a property, used with a specified measurement instrument, whereby it provides indications for one or more measurands such that the indications of each measurand are independent of other measurands or other quantities being investigated (Fig. 4) [1].

#### *Example*

If the real blood flow is stable at 5.0 L/min (as measured in the example in 5.1), and if, at the same time, the indications of a measuring device change from 5.0 to 6.0 L/min while blood pressure is increasing, the tested measuring device is not selective and may be dependent on the blood pressure.

### Resolution

**Fig. 5:**
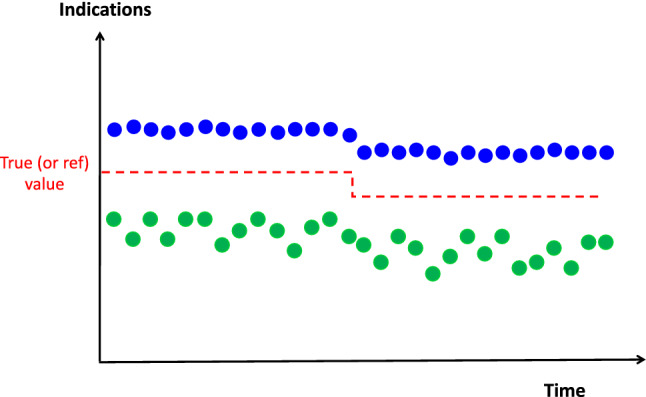
Schematic representation of the resolution. The blue indications show a systematic overestimation of the measurand (bias) and small random error allowing perceiving a small change of the measurand (high resolution). The green indications show a systematic underestimation of the measurand (bias) and high random error hiding a small change of the measurand (low resolution)

The **resolution** is the smallest change in a measurand that causes a perceptible change in the corresponding indication [1]. The concept of resolution is linked to the **discrimination threshold**, the largest change in the measurand that causes no detectable change in the corresponding indication, and to the **dead band**, which is the maximum interval through which a measurand can change in both directions without producing a detectable change in the corresponding indication [1]. Resolution may be linked to the physical granularity of the measurand (pixels, bits, quanta) often coming from the digitalization, but for most physiologic signals the smallest change in a measurand is limited by the standard error of the mean (SEM) of the corresponding indication. The change in the indication could be due to random errors. The SEM is proportional to the variability and to the number (n) of the elementary measurements used to display the indication $2{\rm{~SEM~}} = {\rm{~}}2\sigma {\rm{~}}/{\rm{~}}\sqrt n$. Therefore, resolution, discriminating threshold, and dead band are linked to the random errors of elementary measurements (instrumental precision). A prescribed resolution can be reached by decreasing the random error, or by averaging more elementary measurements to give an indication (Fig. 5). The concept of least significant change (2$\sqrt{2}$ SEM) that can be considered as statistically significant is linked to resolution.

#### *Example*

A blood flow measuring instrument connected to a bench giving a constant flow of 10 L/min provides the elementary measurements every second with a mean value = 10 L/min and a variability σ = 5 L/min. If an indication is to be given by the measuring instrument every second, the smallest perceptible change in the bench signal must exceed 10 L/min to be indicated (2SEM = 2σ/${\sqrt{n}}$ = 10/1). If less, the change in the indication could be due to noise (random errors). Then, the resolution would be 10 L/min. If the manufacturer wanted to reach a resolution of 1 L/min, there were only two solutions: first decreasing the noise (variability) from 5 to 0.5 (2 σ/$\sqrt{n}$ = 1/1), second increasing the number of measurements from 1 to 100 (2 σ /$\sqrt{n}$ = 10/10), therefore giving an indication only every 100 s.

### Step response time

**Fig. 6:**
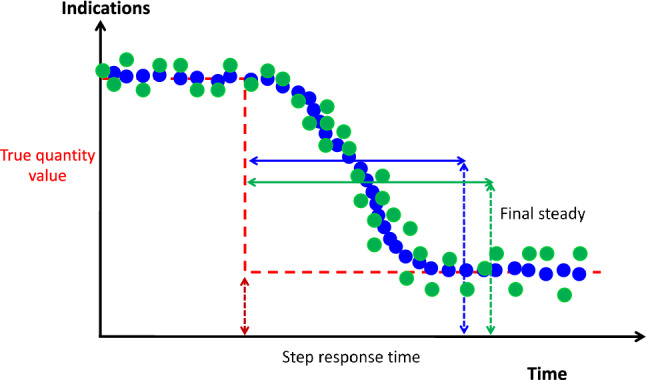
Schematic representation of the step response time. The indications from the test device in blue have higher precision than indications in green allowing a faster identification of the final steady state from which the step response time is derived

The **step response time** is the duration between the instant when a measurand is subjected to an abrupt change and the instant when the corresponding indication of a measuring instrument settles within specified limits around its final steady value (Fig. 6) [1]. The way by which the final steady value is determined may be different: for example, the inflection point between two regression curves, or the first point of a flat curve slope, or the first point where the σ becomes below specified limits. Step response time is also linked to the measurement precision since low precision increases the number of indications needed to establish the final steady state.

#### *Example*

If the real blood flow changes from 5.0 to 6.0 L/min in 10 s (as measured in the example in 5.1), and if the indications of a measuring device changed from 5.0 to 6.0 L/min, in 10 min, the step response time of the tested measuring device is close to 10 min.

### Stability

The **stability** is the property of a measuring instrument, whereby its metrological properties remain constant in time [1]. An **instrumental drift** is a continuous or incremental change over time of the indication due to change in at least one metrological property.

#### *Example*

If the real blood flow (as measured in the example in 5.1) is 5.0 L/min, and if the indications of a measuring device changed from 5.0 to 6.0 L/min within a certain time period (e.g. 12 h), the tested measuring device is not stable.

### Maximum permissible measurement error or limit of errors

The **maximum permissible measurement error (or limits of errors)** is the extreme value of measurement error permitted by specifications or regulations for a given measurement, measuring instrument, or measuring system [1]. The term **tolerance** (not defined in the VIM), should not be used to designate the maximum permissible error [1]. Tolerance most often includes the true value ± the maximum permissible error of a fixed physical property.

#### *Example*

In the preceding example, if the clinical requirements allow a maximum permissible error of 20%, the instability becomes unacceptable after 12 h and the device needs recalibration.

## Measurement standards (etalon)

Any measurement requires a **measurement standard (etalon)**, which is the embodiment of the definition of a given quantity, with stated quantity value and associated measurement uncertainty, used as the reference [1]. This definition shows that the uncertainty with the measurement standard contributes to the combined measurement uncertainty since values that result from the measurement process are in reality ratios between the measured values and the measurement standard, expressed in the same units. In November 2018, the BIPM has changed the definitions of the international standards. All definitions are now based on atomic constants to minimize uncertainties [5]. These changes came into force on May 20th, 2019 [5].

### *Example*

The kilogram was defined until now by the mass of a cylinder alloy (90% platinum and 10% iridium) manufactured in 1889, stored at the BIPM, with official copies sent in 40 national metrologic centers worldwide. Although carefully stored, these etalons diverge from the original by ≈ 50 μg per century. Therefore, the kilogram is now defined by the Planck constant set at = 6.62607015 × 10 ^− 34^ m^2^ kg s^− 1^ and practically obtained from a Kibble balance.

### Calibration

**Fig. 7:**
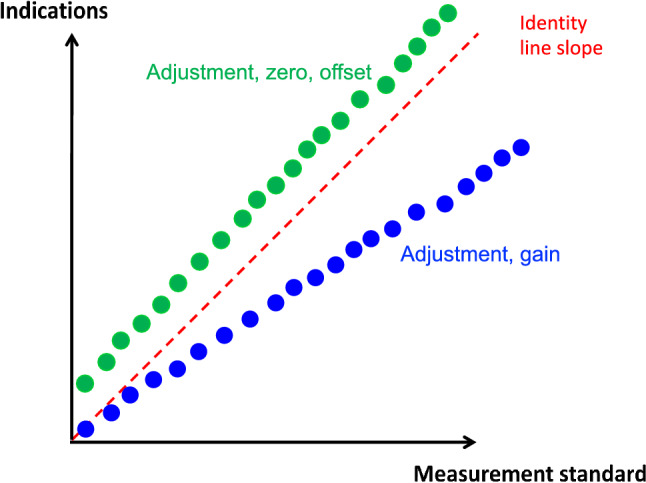
Schematic representation of two examples of adjustments required for a measuring system. The green indications show an offset and the blue indications show an insufficient gain

A measurement standard is the prerequisite of any **calibration**, which is the operation that, in a first step (in specified conditions) establishes a relation between a device indication and the corresponding quantity values provided by a measurement standard (with known uncertainty) and, in a second step, uses this information for obtaining a measurement result (with appropriate units) from an indication [1]. Strictly speaking, calibration is just a comparison [1]. However, in general use, the term calibration also refers to a second step that is using these initial steps for (1) the verification that the test device meets the prescribed standards, and if not, (2) for the **adjustment of a measuring system**, sometimes improperly called “auto-calibration”, which is the set of operations (zero, offset, and span or gain adjustment) carried out on a measuring system so that it provides prescribed indications corresponding to given values of a measurand (Fig. 7) [1].

#### *Example*

If the real blood flow is 4.0, 5.0, and 6.0 L/min during a given maneuver, and if the corresponding indications of a measuring device are 5.0, 6.0, and 7.0 L/min, the measuring device needs a recalibration (zero and offset). If the corresponding indications of another measuring device are 4.0, 4.5, and 5.0 L/min, the second measuring device needs a recalibration (offset and gain).

### Metrological traceability

The **metrological traceability** is the property of a measurement result whereby the result can be related to a reference through an unbroken chain of calibrations, each contributing to the measurement uncertainty [1]. An unbroken chain of calibrations means that SI units are determined. The working instrument is then compared (calibrated) with the best practically available reference method. This reference method is compared to a higher standard (a standard with less uncertainty) again and again, and the chain is documented through calibration certificates.

## Conclusion

In perioperative and intensive care medicine, the metrological requirements for measurements (facts) and measuring instruments (methods) are poorly defined. One of the reasons may be the lack of consensus among physicians and scientific societies on which are the minimum quality criteria. Full transparency is needed in the validation of new measuring devices [6]. Adopting the same understandings and definitions among physicians and scientists is obviously the first step.

## Abbreviations

VIM: International Vocabulary of Metrology
σ: Standard deviation
σ^2^: Variance
SEM: Standard error of the mean
BIMP: International Bureau of Weights and Measures (Bureau International des Poids et Mesures)
SI: International standard
CO: Cardiac output
BP: Blood pressure
